# The Effects of Cerebral Perfusion Pressure Change on Acute Intraoperative Brain Herniation in Patients With Severe Traumatic Brain Injury

**DOI:** 10.7759/cureus.85890

**Published:** 2025-06-12

**Authors:** Buqing Liang, Shuai Hu, Liang Zhang, Likun Yang, Yuhai Wang

**Affiliations:** 1 Neurosurgery, Loma Linda University Medical Center, Loma Linda, USA; 2 Neurosurgery, 904 Hospital of the People's Liberation Army Joint Logistic Support Force, Wuxi, CHN

**Keywords:** brain herniation, cerebral perfusion pressure, encephalocele, external ventricular drain, intracranial pressure, intracranial pressure monitor, risk factors, traumatic brain injury

## Abstract

Objective

This study examines how rapid changes in cerebral perfusion pressure (CPP) affect acute intraoperative brain herniation (AIBH) in patients with severe traumatic brain injury (sTBI).

Materials and methods

This single-institute retrospective study analyzed patients with sTBI (Glasgow Coma Scale (GCS) ≤ 8) who underwent surgical intervention from January 2017 to January 2019. An external ventricular drain (EVD)/intracranial pressure monitor (ICPm) was placed in all patients preoperatively. Intracranial pressures (ICPs) before the surgery at the time of both craniotomy and durotomy were recorded. CPP, calculated as mean arterial pressure (MAP) - ICP, was recorded as initial (CPPi), at the time of craniotomy (CPPc), and at the time of durotomy (CPPd). Changes in CPPc and CPPd were calculated as (CPPc - CPPi)/CPPi and (CPPd - CPPc)/CPPc, respectively. The effects of the change in CPP on AIBH were categorized into three groups according to the significance of the changes and were analyzed using SPSS version 22.0 (IBM Corp., Armonk, NY).

Results

A total of 98 cases were recruited, including 77 (78.57%) male patients and 21 (21.43%) female patients. Age ranged from 19 years old to 77 years old, with a mean/median of 49.01/50.50 years old. The changes in CPP at the time of both craniotomy and durotomy were all significantly related to AIBH. Changes in CPP were classified into three groups: (1) both were <50%, (2) at least one was ≥50% but <100%, and (3) at least one was ≥100%. The incidence rates of AIBH in the three groups were 27.1% (13/48), 61% (25/41), and 77.8% (7/9), respectively. Significant differences were also observed between each two groups (p<0.05).

Conclusion

Changes in CPP appear to be related to acute intraoperative brain herniation in patients with severe traumatic brain injury undergoing surgical intervention.

## Introduction

Severe traumatic brain injury (sTBI) patients with a Glasgow Coma Scale (GCS) of ≤8 tend to have extremely high morbidity and mortality rates (MMRs), with an associated permanent disability rate of 100% [1]. Open craniotomy/craniectomy (OC) has been proven to be an effective method of lowering refractorily elevated intracranial pressure (ICP) [2]. Even so, devastating prognoses are still commonly seen, especially in those with acute intraoperative brain herniation (AIBH) during OC [3]. The prevention of such has been a key component leading to a successful intervention and decreased MMRs. Attempting to prevent and manage AIBH effectively, studies have been conducted on the risk factors of AIBH in OC worldwide, including remote skull fracture/hematoma, preoperative hypoxia/ischemia, diffuse brain edema, preoperative brain herniation, and continuous intracranial pressure (ICP) monitoring [4,5]. However, during OC, cerebral perfusion pressure (CPP) might be a neglected index, which is more controllable. So far, to the best of our knowledge, there is no report on the relationship between changes in CPP and AIBH during OC. Our study is designed to explore this topic.

## Materials and methods

This is a single-institute, retrospective study investigating the correlation between changes in CPP and AIBH in sTBI patients who underwent OC during a two-year period from January 2017 to January 2019. The study was approved by the hospital’s institutional review board. Due to the emergency of the cases, consent forms were waived except in those cases where patients’ family members were available.

Inclusion criteria

All sTBI patients who underwent OC were recruited. Cases with simple depressive skull fractures and pure epidural hematomas were excluded, considering their low risk of brain herniation. All patients received an external ventricular drain (EVD) or intracranial pressure monitor (ICPm) and arterial line placement preoperatively. The ICP and mean arterial pressure (MAP) were measured constantly and simultaneously. The diagnostic criteria for AIBH were defined as progressive brain herniation that extended beyond the borders of the skull opening that could not be controlled with external ventricular drainage, hyperventilation, and/or mannitol/diuretics. Meanwhile, one of the following two criteria must be met: (1) brain tissue extending more than 1 cm over the skull opening border to be compressed by the latter with decreased or disappeared pulsation that could not be reduced, and (2) although the herniation of brain tissue was within 1 cm of the border of the skull opening, the trend remained progressive, which required emergent surgical intervention as there was either intraoperative computed tomography (CT) or ultrasound evidence of delayed hematoma. Exclusion criteria included the following: (1) AIBH secondary to an insufficient skull opening, (2) AIBH that could be reduced intraoperatively with no delayed or expanding hematoma on postoperative CT imaging, and (3) AIBH secondary to patients’ positioning.

Risk factors

The following risk factors were analyzed: age, gender, admission Glasgow Coma Scale (GCS), admission to the operation period, and changes in CPP at the time of craniotomy (Cc) and durotomy (Cd).

Surgical intervention

All patients were treated according to the Chinese Guideline for Management of Severe Traumatic Brain Injury. All operations were performed by attending neurosurgeons who specialized in brain trauma. Preoperative CT imaging was utilized for approach design. Frontal EVD (Codman 82-6653, Johnson & Johnson, New Brunswick, NJ) through Kocher’s point, aiming at the foramen of Monro, was preferred in all cases unless there was any contraindication. For difficult cases or failed attempts, it was left in place as an intraparenchymal ICPm. The initial ICP was recorded, as well as concomitant MAP via the arterial line. For patients with brain herniation signs or symptoms, cerebrospinal fluid (CSF) was immediately drained to lower the ICP. In some cases, a swift mini-subtemporal craniotomy and durotomy would be performed to evacuate hematoma and lower ICP [6]. Under continuous monitoring of ICP and MAP, craniotomy and durotomy were performed. CPP, calculated as MAP - ICP, was recorded as initial (CPPi), at the time of craniotomy (CPPc), and at the time of durotomy (CPPd). Changes in CPPc and CPPd were calculated as (CPPc - CPPi)/CPPi and (CPPd - CPPc)/CPPc, respectively. The changes were then subdivided into three categories: (1) both were < 50%, (2) at least one was ≥50% but <100%, and (3) at least one was ≥100%.

Statistical analysis

Multivariate logistic regression models were utilized. A p-value of less than 0.05 was considered significant. All statistical analyses were performed using SPSS version 22.0 (IBM Corp., Armonk, NY).

## Results

A total of 98 cases were recruited for data analysis, of which 77 (78.6%) were male patients and 21 (21.4%) were female patients (Table 1). Age ranged from 19 to 77 years old, with a mean/median of 49.01/50.50 years old. On admission, 46 (46.9%) cases fell into the GCS range of 3-5, and 52 (53.1%) cases’ GCS were 6-8. The causes of trauma included 72 (73.5%) traffic accidents, 15 (15.3%) falls from height, five (5.1%) ground-level falls, four (4.1%) heavy mass strikes, and two (2.0%) assaults. EVD was successfully placed into the lateral ventricle at first attempt in 67 (68.4%) cases and in 24 (24.5%) cases after adjustment. In seven (7.1%) cases where no CSF could be obtained, the catheter was used as an intraparenchymal ICPm. The time interval from the accident to operation ranged from one hour to 48 hours, with a mean/median of 2.78/1.5 hours. Forty-five (45.9%) cases had AIBH, while 53 (54.1%) were negative for AIBH. The CPPi ranged from 5.00 to 97.00 mm Hg, with a mean/median of 47.71/46.00 mm Hg. CPPc ranged from 10.00 to 87.00 mm Hg, with a mean/median of 56.85/57.50 mm Hg. CPPd ranged from 19.00 to 96.00 mm Hg, with a mean/median of 65.89/68.00 mm Hg. The Cc ranged from -46.51% to 300.00%, with a mean/median of 28.99%/21.34%. The Cd ranged from -70.67% to 510.00%, with a mean/median of 34.21%/7.52% (Table 2). There were 48 (49.0%) category 1 cases, 41 (41.8%) category 2 cases, and nine (9.2%) category 3 cases. In each category, the AIBH rate was 27.1% (13/48), 61% (25/41), and 77.8% (7/9), respectively (Table 3).

**Table 1:** Patient characteristics GCS: Glasgow Coma Scale, MVC: motor vehicle collision, FFH: fall from height, GLF: ground-level fall, EVD: external ventricular drain, CSF: cerebrospinal fluid, ICPm: intracerebral pressure monitor, AIBH: acute intraoperative brain herniation

Characteristics	Number (%)
Sex	
Male	77 (78.6%)
Female	21 (21.4%)
GCS	
3-5	46 (46.9%)
6-8	52 (53.1%)
Causes	
MVC	72 (73.5%)
FFH	15 (15.3%)
GLF	5 (5.1%)
Strikes	4 (4.1%)
Assault	2 (2.0%)
EVD with CSF	
1st attempt	67 (68.4%)
2nd/3rd attempt	24 (24.5%)
EVD as ICPm	7 (7.1%)
AIBH	
Yes	45 (45.9%)
No	53 (54.1%)

**Table 2:** Characteristics of the surgical intervention Time interval: from admission to operation, CPPi: initial cerebral perfusion pressure, CPPc: cerebral perfusion pressure at craniotomy, CPPd: cerebral perfusion pressure at durotomy, Cc: cerebral perfusion pressure change at craniotomy, Cd: cerebral perfusion pressure change at durotomy

Characteristics	Range	Mean	Median
Time interval (hours)	1-48	2.78	1.5
CPPi (mm Hg)	5-97	47.71	46
CPPc (mm Hg)	10-87	56.85	57.50
CPPd (mm Hg)	19-96	65.89	68.00
Cc (%)	-46.51 - 300.00	28.99	21.34
Cd (%)	-70.67 - 510.00	34.21	7.52

**Table 3:** Comparison of clinical characteristics among different change categories Category 1: Both CPPc and CPPi were < 50%. Category 2: At least one change was ≥50% but <100%. Category 3: At least one change was ≥100%. POBH: preoperative brain herniation with a dilated pupil or pupils, AIBH: acute intraoperative brain herniation, SD: standard deviation, GCS: Glasgow Coma Scale Multivariate logistic regression models were utilized for analysis.

Variable	Category 1	Category 2	Category 3
Cases (number, %)	48 (49.0%)	41 (41.8%)	9 (9.2%)
Age (years, mean±SD)	50.1±13.1	47.5±13.5	49.8±12.6
Sex			
Female (number, %)	11 (22.9%)	10 (24.4%)	0 (0%)
Male (number, %)	37 (77.1%)	31 (75.6%)	9 (100%)
GCS (mean±SD)	6.0±1.7	5.4±1.6	4.7±1.6
Admission to OR interval (hours)	3.7±0.4	1.8±1.0	1.7±0.5
POBH (number, %)	16 (33.3%)	22 (53.7%)	8 (88.9%)
AIBH (number, %)	13 (27.1%)	25 (61.0%)	7 (77.8%)

Changes in CPP at both craniotomy and durotomy were significantly related to AIBH (p<0.005). The odds ratios (OR) (95% confidence interval) were 2.137 (1.524-8.716) and 8.909 (5.741-20.059), respectively. There were also significant differences between each category group (p<0.05) (Table 4).

**Table 4:** Multivariate logistic regression models for acute intraoperative brain herniation related to Cc and Cd Cc: cerebral perfusion pressure change at craniotomy, Cd: cerebral perfusion pressure change at durotomy, OR: odds ratio, 95% CI: 95% confidence interval

	OR	95% CI	p-value
Cc	2.137	1.524-8.716	<0.005
Cd	8.909	5.741-20.059	<0.005

## Discussion

The management of traumatic brain injury has been a challenging topic around the globe, and many patients with such disease undergo emergent neurosurgical intervention. Acute intraoperative brain herniation remains one of the most onerous complications that leads to a detrimental prognosis. As reported in the literature, remote skull fracture/hematoma, preoperative ischemia/hypoxia, diffuse brain swelling, and preoperative brain herniation are all closely related to AIBH [4,7,8]. How to effectively prevent AIBH and improve outcomes constitutes the goal of this study.

Hematoma

As reported, most of the remote hematomas secondary to decompressive surgery have been epidural hematomas, and an underlying fracture would usually be demonstrated [9]. Injuries to the middle meningeal artery or its branches, arachnoid granulations, or venous sinuses will result in the formation of a hematoma and increased ICP. The latter, to a certain extent, restrains the expansion of hematoma. In these cases, the tamponade effect would be released or totally effaced once craniotomy or durotomy is performed. With the decreased extravascular pressure, rapid vessel filling secondary to increased blood flow will cause rapid blood extravasation from the originally injured blood vessels, pushing the brain toward the contralateral side with resultant herniation. Cepeda et al. found that traumatic intracerebral hemorrhage growth was significantly related to craniectomy [10]. Intraoperative ultrasound is a useful tool for the detection of contralateral subdural hematoma or intraparenchymal hemorrhage. However, a negative result could not totally rule out such. With a low suspicion of diffuse brain swelling, an immediate postoperative head CT should be obtained to evaluate any delayed formation of hematoma [7]. As seen in our case presentation, rapid evacuation of the epidural hematoma at the contralateral side restores the brain to its normal position and provides the patient with a better chance of a good outcome. Identification of bone fractures/hematoma on the preoperative CT imaging and correction of coagulopathy are also crucial in the management [1,11].

Brain swelling and herniation

Various severities of brain swelling are always present in TBI patients [12]. Radiographically, loss of gray-white junction, effacement of ventricles, and obliteration of normal cisterns are presentations indicating diffuse brain swelling [13]. Preoperative brain herniation itself is an indication for acute surgical intervention in sTBI patients. Clinical presentations are most prominent for mydriasis secondary to cranial nerve III palsy. Gurer et al. found that in patients with preoperative brain herniation secondary to extra-axial hematoma who underwent surgical intervention, intraoperative brain swelling was highly correlated with poor prognosis (p<0.001) [4]. Hypotension and hypoxemia could exist either independently or simultaneously in these sTBI patients, leading to brain tissue ischemia, thus worsening outcome [8]. In our study, persistent hypoperfusion during surgery with a CPP < 60 mm Hg is also significantly related to AIBH (66.7%, 8/12). With increased ICP and decreased MAP, the CPP would be further compromised, resulting in more hypoxia and brain swelling, forming a vicious circle, and finally AIBH. Even worse, the kinking of draining veins at the edge of the skull opening in AIBH further exacerbates brain swelling [14]. Early diagnosis and treatment of hypovolemia and pulmonary injuries with cessation of bleeding, infusion of fluid, transfusion of blood, and support of ventilation, etc., to avoid hypotension and hypoxia is critical. Medically, mannitol, hypertonic saline, hyperventilation, and brain suppression are commonly used for alleviating brain edema. Surgically, controlled decompression technique has been proposed by different authors, which seems to lower the incidence of intraoperative brain swelling and result in better outcomes [6,15].

ICP versus CPP

In normal adults, the ICP ranges from 5 to 15 mm Hg and CPP from 50 to 70 mm Hg [16,17]. In sTBI patients, the fourth edition guideline from the Brain Trauma Foundation recommends treating ICP > 22 mm Hg and target CPP between 60 and 70 mm Hg [18]. Acute OC has been proven to effectively control increased ICP refractory to medical management in sTBI patients [19]. Under continuous monitoring, the ICP tends to trend by the following pattern: first drop at craniotomy, second drop at durotomy, and third drop after hematoma evacuation. In general, after these three steps, the ICP would gradually return to the normal range. Meanwhile, a rebound uptrending ICP requires intensive investigation. The two most common underlying mechanisms include acute brain swelling and the formation of a new delayed hematoma, as discussed above. The damaged autoregulation of intracranial blood vessels and decreased brain compliance in sTBI patients indicate that relying solely on the absolute ICP value for management is probably not sufficient, and CPP seems to be a better index. Rosner et al. proposed CPP-guided management in sTBI patients as it is most important in maintaining CBF and is most amenable to physicians [20]. Pressure reactivity index (PRx) has been utilized to estimate the optimized CPP in sTBI patients [21,22]. However, an applicable algorithm is yet to be validated. Recently, studies showed that guidelines-based management of CPP monitoring in sTBI patients decreased two-week mortality [18,23]. Eun et al. also discovered that for patients with TBI who received decompressive craniectomy (DC), if postoperative ICP was maintained at <25 mm Hg, targeted CPP might be lowered to 35 mm Hg with similar mortality to patients with CPP > 60-70 mm Hg without DC [24].

Change in CPP

During the process of surgical intervention, the most dramatic drops of ICP transpire at stages of craniotomy and durotomy. With a constant MAP, the CPPs would increase drastically at these two points, causing unintended damage to the autoregulation system of the intracranial blood vessels as a result of vasogenic edema due to the imbalance of Starling forces at the capillaries [16]. Meanwhile, the whole intracranial blood volume tends to increase due to the lack of prompt venous system opening, exacerbating AIBH. On the other hand, CPP at times could present a negative growth during these two points. The causes for this could be multifaceted: (1) a bounce of ICP secondary to brain swelling/remote hematoma that exceeds the transient increase of MAP and (2) rapid blood loss from craniotomy/intracranial bleeding and/or deep anesthesia, leading to a drop in MAP, which exceeds the decrease in ICP. Our study laid emphasis on the changes in CPP at craniotomy and durotomy, which revealed a significant relationship between both and AIBH, and the higher the changes, the more likely the AIBH would be. We thus advocate for CPP control during surgical intervention, especially at craniotomy and durotomy. Methods that could be applied mainly include MAP adjustment and controlled decompression. Techniques for the latter include slow removal of the bone flap and staged durotomy [6,15]. Meanwhile, a negative growth of CPP should be avoided to prevent brain ischemia and further damage.

Limitations

The validity of this study may be restrained by its intrinsic single-institute background. There are some outliers, such as the 48-hour time interval from admission to operation and a low CPPi of 5 mm Hg. We kept these numbers for data integrity and transparency. More rigorous inclusion criteria may be used in future studies. The diagnosis of AIBH was subjectively defined by the operating neurosurgeon, which may introduce bias. Category 3 includes only nine cases, which could be underpowered. A study including multiple institutions and a higher number of cases could overcome these potential limitations. Additionally, it is reasonable to anticipate higher risks of brain swelling and herniation with higher ICPs, which could be investigated in future studies as a potential confounder.

## Conclusions

Changes in cerebral perfusion pressure appear to be related to acute intraoperative brain herniation in patients with severe traumatic brain injury undergoing surgical intervention. Further studies focusing on causal relationships and optimizing these factors to improve outcomes are warranted.
